# Nanocomposite Electrode of Titanium Dioxide Nanoribbons and Multiwalled Carbon Nanotubes for Energy Storage

**DOI:** 10.3390/ma16020595

**Published:** 2023-01-07

**Authors:** Mohammad BinSabt, Mohamed Shaban, Ahmed Gamal

**Affiliations:** 1 Chemistry Department, Faculty of Science, Kuwait University, P.O. Box 5969, Safat 13060, Kuwait; 2 Department of Physics, Faculty of Science, Islamic University of Madinah, P.O. Box 170, Madinah 42351, Saudi Arabia; 3 Nanophotonics and Applications Laboratory, Physics Department, Faculty of Science, Beni-Suef University, Beni-Suef 62514, Egypt

**Keywords:** nanocomposite, supercapacitor, TiO_2_, CNTs, energy storage

## Abstract

TiO_2_ is one of the most investigated materials due to its abundance, lack of toxicity, high faradaic capacitance, and high chemical and physical stability; however, its potential use in energy storage devices is constrained by its high internal resistance and weak van der Waals interaction between the particles. Carbon nanotubes are especially well suited for solving these issues due to their strong mechanical strength, superior electrical conductivity, high electron mobilities, excellent chemical and thermal stability, and enormous specific nanoporous surface. The hydrothermal approach was followed by chemical vapor deposition to produce a network composite of titanium dioxide nanoribbons (TNRs) and multi-walled carbon nanotubes (MWCNTs). The nanocomposite was characterized using a variety of methods. One phase of TiO_2_-B nanoribbons has porous pits on its surface, and MWCNTs are grown in these pits to produce a network-like structure in the nanocomposite. With a two-electrode supercapacitor configuration, the TNR/CNT gave a gravimetric capacitance of 33.33 F g^−1^, which was enhanced to 68.18 F g^−1^ in a redox-active electrolyte containing hydroquinone (HQ). Additionally, the areal capacitance per footprint was increased from 80 mF cm^−2^ in H_2_SO_4_ to 163.63 mF cm^−2^ in H_2_SO_4_/HQ. The TNR/CNT supercapacitor has superior cyclic stability than the previously reported TiO_2_-based electrodes, with 97.5% capacitance retention after 5000 cycles. Based on these results, it looks like the TNR/CNT supercapacitor could provide portable electronic power supplies with new ways to work in the future.

## 1. Introduction

Portable smart devices could benefit from supercapacitors because of their fast discharge speed, long cycle life, and high-power density, among other advantages. In terms of energy storage, supercapacitors are divided into electrochemical double-layer capacitors with higher power density linked to the electrode surface area and pseudocapacitors with higher energy density linked to faradaic redox [1]. High-performance electrode materials for supercapacitors have mostly been developed by modifying and blending pseudocapacitive materials such as transition metal oxides and conductive polymers with carbonaceous materials from double-layer capacitors [2,3]. As electrodes for supercapacitors, carbon nanotube electrodes have proven to retain the remarkable qualities of individual carbon nanotubes while also exhibiting good mechanical properties and structural stability [4,5]. This study also examined the properties of titanium dioxide (TiO_2_), which has a high faradaic capacitance and is chemically and physically stable [6]. Despite this, TiO_2_’s high internal resistance prevents it from being used in an energy storage device. Due to the weak van der Waals interaction between the particles, TiO_2_ has not yet been successfully used as an electrode in a supercapacitor, but it can be combined with other materials, particularly carbon-based materials. As shown in Table 1, previous studies on TiO_2_/activated carbon, TiO_2_/carbon nanotubes, and reduced graphene oxide/TiO_2_ nanobelt composites revealed that the combination of conductive nanocarbon and TiO_2_ was an efficient solution [7,8,9,10,11,12,13,14,15]. Selvakumar and Bhat developed TiO_2_/activated carbon nanocomposite electrodes, which have a specific capacitance of 122 F/g at current densities of 2, 4, 6, and 7 mA/cm^2^ [7]. A PANI/TiO_2_/GO composite with high specific capacitance—1020 F/g at 2 mV/s and 430 F/g at 1 A/g—was made by Su et al. [8]. The rGO-TiO_2_ nanobelts and nanoparticles made by Xiang et al. had specific capacitances of 225 and 62.8 F/g, respectively. Ramadoss et al. produced an rGO/TiO_2_ NR/rGO electrode with 114.5 F/g at a scan rate of 5 mV/s that maintained more than 85% of its initial capacitance after 4000 cycles [10]. Ramadoss and Kim used a microwave-assisted technique to produce a graphene–TiO_2_ hybrid nanostructure with a specific capacitance of 165 F/g at a scan rate of 5 mV/s in a 1 M Na_2_SO_4_ solution and retention of 90% specific capacitance after 5000 cycles [11]. High specific and interfacial capacitances, of 329 F/g and 52 mF/cm^2^ at a scan rate of 5 mV/s were achieved by coating TiO_2_ nanodots on MWCNTs using a binder-free method developed by Sankapal et al. [12]. Using a sacrificial template technique, Ke et al. produced a 3D carbon/TiO_2_/rGO composite with a specific capacitance of 23.6 mF/cm^2^ [13]. In a 0.5 M H_2_SO_4_ electrolyte, the TiO_2_/CNT hybrid produced by Yan et al. using the sol–gel technique exhibits a specific supercapacitance of 145 F/g [14]. By co-electrochemically reducing functionalized MWCNTs and GO onto TiO_2_NTs/Ti, Faraji created a 3D R(fMWCNT-GO)/TiO_2_NTs/Ti electrode with a specific capacitance of 600 F/g at 12 A/g in 1 M H_2_SO_4_ and a long cycle life of 90% capacitance retention over 500 cycles [15]. Despite previous research, it is urgent to create TiO_2_/carbon-based nanocomposite electrodes for supercapacitors that are less expensive, have a high yield and surface area, and are more stable.

The hydrothermal approach was used in this study to achieve the controllability of TiO_2_ structures in a simple, low-cost method while also significantly increasing the energy storage capacity of the electrode materials. Metal carbides will be used more in energy storage devices as a result of this. The TiO_2_ nanoribbon acts as a substrate for the chemical vapor deposition (CVD) of carbon nanotubes on its surface. In various solutions, electrochemical impedance spectroscopy (EIS), cyclic voltammetry (CV), and galvanostatic charge/discharge (GCD) were used to measure the nanocomposite performance. Their capacity, specific power, and specific energy were also investigated to evaluate how well they will operate over 5000 cycles of reusability.

## 2. Experimental Section

### 2.1. Materials

Loba Chemie (Mumbia, India) provided the TiO_2_ powder (Loba Chemie, India, CAS No. 13463-67-7), Fe (NO_3_)_3_·9H_2_O (Loba Chemie, India, CAS No. 7782-61-8), Co(NO_3_)_2_·H_2_O (Loba Chemie, India, CAS No. 10026-22-9), and Al(NO_3_)_3_·9H_2_O (Loba Chemie, India, CAS No. 7784-27-2). Scharlab (Barcelona, Spain) supplied HCl (36.6%). SDFCL (Mumbia, India) supplied H_2_SO_4_ (98%) and HNO_3_ (69%). Deliveries from ADWIC (Cairo, Egypt) included commercial C_2_H_4_ gas, NH_4_OH (32%) and NaOH.

### 2.2. TNRs/CNTs Nanocomposite Fabrication

TNRs were made using an alkaline hydrothermal process. In 400 mL of 10 M NaOH, 4 gm of TiO_2_ powder was added and stirred for 30 min. In a 1 L autoclave, the resulting solution was poured. The autoclave was then placed in a 170 °C oven for 24 h. The result was then filtered before being washed with 0.1 M HCl and distilled water. Finally, the white powder was dried for 4 h at 80 °C and calcined for 2 h at 450 °C [16].

The TiO_2_ nanoribbons were mixed with Fe (NO_3_)_3_·9H_2_O, Co (NO_3_)_2_·6H_2_O, and Al (NO_3_)_3_·9H_2_O in 100 mL of distilled water under stirring, with mass ratios of 10 TiO_2_:20 Fe:20 Co:50 Al_2_O_3_. By slowly drizzling in drops of ammonia solution, the pH level rose to 8 and the precipitation occurred. The solution was filtered and rinsed with distilled water. The solution was aged for 2 h at room temperature. To eliminate excess nitrate, the product was first dried for 4 h at 80 °C and then calcined for 4 h at 450 °C [17].

The functionalized titanium nanoribbons were used as a catalyst for the formation of CNTs using tubular chemical vapor deposition (CVD). The carbon source was C_2_H_4_, and the carrier gas was N_2_. C_2_H_4_:N_2_ was a 1:10 *v*/*v* ratio. The CNTs were grown for 50 min at 700 °C [18]. For 6 h at 120 °C, we heated the CVD-prepared product in a round bottom flask to dilute it with H_2_SO_4_: HNO_3_ (1:3). Next, the powder was cleaned and dried at 80 °C for 4 h using distilled water [19,20]. A total of 1 g of the composite is expected to cost between $5 and $6.

### 2.3. Characterization of the Produced Nanomaterials

X-ray diffraction (XRD; PANalytical, Warsaw, Poland) using Cu K α radiation (=1.5406 Å) at 45 kV and 40 mA validated the crystal structure of the CNTs and TNRs/CNTs. A transmission electron microscope (TEM) was used to examine the morphologies of the produced nanocomposites (JEOL JEM-2100 TEM, Tokyo, Japan).

### 2.4. Supercapacitor Manufacturing Processes

A total of 20 mg of active material powder and 50 µL of Nafion were dispersed in 300 µL of ethanol and then blended into a slurry in a smaller agate mortar. To make a homogenous catalyst ink, the mixture was agitated for 12 h. On the Au electrode, two identical slurries of about 50 µL (3 mg) were placed (1 cm^2^). Two sheets of filter paper, on the other hand, were dipped in electrolytes, which may be either 1 M H_2_SO_4_ or a mixture of 1 M H_2_SO_4_ and 0.4 M hydroquinone (HQ) (H_2_SO_4_/HQ). After that, a sheet of filter paper was placed between the two electrodes as a separator.

### 2.5. Electrochemical Analysis

All electrochemical measurements in the two-electrode systems were performed using an electrochemical workstation (CHI 660E; CH Instruments, Austin, TX, USA). Cyclic voltammetry (CV), galvanostatic charge/discharge (GCD), and electrochemical impedance spectroscopy (EIS) were used to make the measurements. The CV experiments were carried out at various scan rates between 0 and 1 V, ranging from 5 to 100 mV s^−1^. The GCD measurements were carried out in a voltage window of 0 to 1 V at 0.2–1 A g^−1^. The EIS spectra were recorded at an open circuit potential of 5 mV AC voltage amplitude and frequencies ranging from 10 mHz to 100 kHz. The tests were all conducted at room temperature.

## 3. Results and Discussions

### 3.1. Characterization of the Produced Nanomaterials

#### 3.1.1. Structural Properties

**Figure 1:**
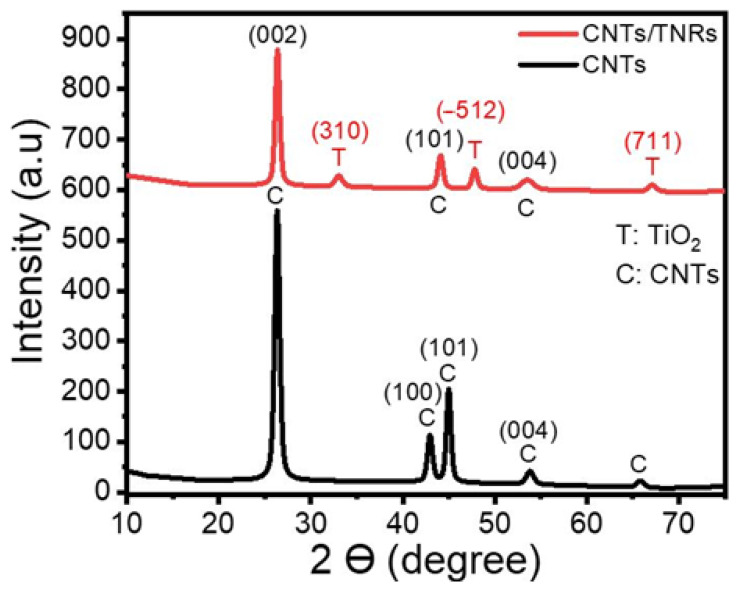
The XRD pattern of a TNR/CNT nanocomposite.

XRD spectroscopy was used to analyze the TNR/CNT nanocomposite and identify its crystal structure. The XRD charts for the nanocomposite CNTs and TNRs/CNTs are displayed in Figure 1. A strong peak associated with XRD diffraction from the (002) plane can be seen on the CNTs chart at 26.32°. In addition, the peaks at 42.91° and 44.94° are connected to the carbon’s in-plane (100) and (101) reflections, respectively, while the peak at 65.74° is connected to the XRD from (004) [21]. 

The XRD pattern of TNRs/CNTs exhibited three unique CNT peaks at 2ϴ = 26.34°, 44.05°, and 53.48°, in the (002), (101), and (004) planes, respectively [17]. Additionally, TiO_2_-B is associated with the peaks in the Figure 1 planes (310), (−512), and (711) at 33.0°, 47.76°, and 67.02° [22,23,24].

The average crystallite size, CS, was determined using the Scherrer equation $\mathrm{CS}=\frac{0.9\;\lambda }{\beta \cos\theta }$ where β is the full width at half maximum (FWHM); θ is the Bragg’s angle in radians; and λ is the X-ray wavelength (CuK_α_ = 0.15405 nm) [25,26]. Additionally, the dislocation density (δ) was determined by applying Williamson and Smallman’s relation, where *N* = 1 represents the minimum dislocation density and δ = $\frac{N}{{\mathrm{CS}}^{2}}$. Using Equation (1), the texture coefficient (TC) was also calculated from the data [25,26].

(1) $$\mathrm{TC}(hkl)=\frac{I_{r}\left(hkl\right)}{\frac{1}{N}\sum I_{r}\left(hkl\right)}$$

where *N* is the number of reflections and I_r_ = $\frac{I\;\left(hkl\right)}{I_{0}\;\left(hkl\right)}$ is the difference between the measured intensity I(*hkl*) and the standard intensity I_o_(*hkl*) for the plane *hkl*.

Table 1 contains the computed and actual values for the CNTs and the nanocomposite’s crystallite sizes (CS), d-spacing, dislocation densities, texture coefficients, and microstrain. Growing CNTs on the surface of TNRs resulted in an increase in the crystallite size of CNTs along (002) from 14.7 nm to 16.8 nm. TNRs typically have crystallite sizes between (−512) and (310) of 15.6 and 9.9 nm, respectively. Based on the values of the texture coefficient in Table 1, the preferred orientations for TiO_2_-B (TNRs) and CNTs were (−512) and (002), respectively. Additionally, the (−512) and (002) planes had lower TNR and CNT dislocation densities than the other planes. This is brought on by the grains along these planes having a higher crystallinity [27]. Table 1 reports the positive microstrain values, which indicate a lattice expansion and relaxation [28]. The CNTs in the pure sample and the composite are reported to have the highest values of microstrain along the (002).

#### 3.1.2. Morphological Analysis

**Figure 2:**
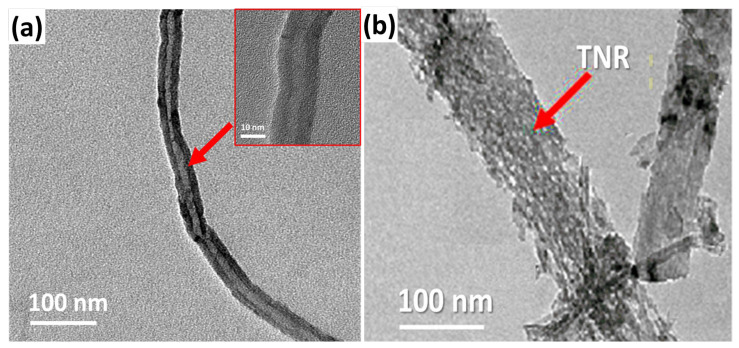
TEM pictures of (**a**) the CNTs and the (**b**) TNR/CNT nanocomposite.

TEM was used to analyze the nanocomposite’s morphology. As illustrated in Figure 2a, the CVD-grown CNTs formed multi-walled carbon nanotubes (MWCNTs), with the inner and outer tubes having widths of 5 to 7 nm and 15 to 17 nm, respectively. TiO_2_ nanoribbons have a structure that is wider, longer, and straighter. The nanoribbons’ typical width ranged from 20 to 200 nm. As illustrated in Figure 2b, these nanoribbons have a dense distribution of nano pits on their surfaces. The nano pits have a diameter ranging from 4 to 8 nm [29]. As seen in the inset figure, the nanoribbon works as a substrate for the growth of carbon nanotubes on its surface.

### 3.2. TNR/CNT Nanocomposite Electrochemical Performance in 1 M H_2_SO_4_

**Figure 3:**
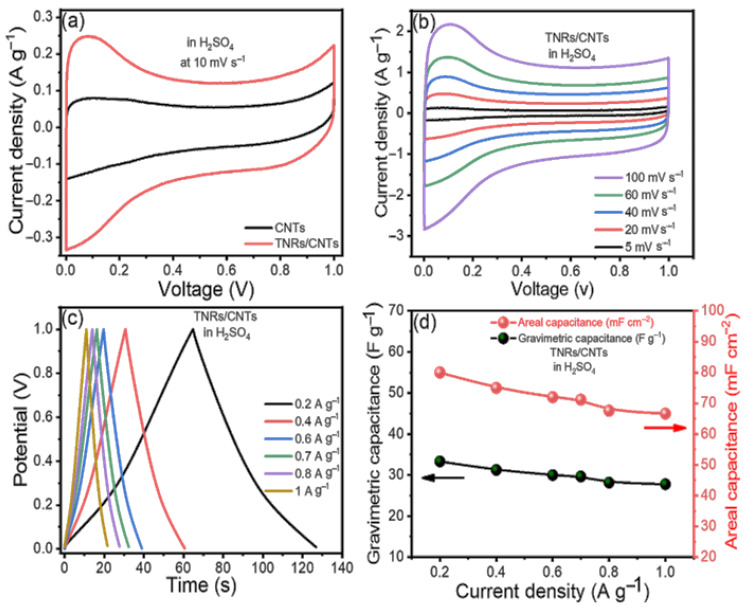
The electrochemical performance of the TNR/CNT electrodes in the H_2_SO_4_ (**a**) cyclic voltammograms (CVs) of the CNTs and TNR/CNTs at a scan rate of 10 mV s^−1^, (**b**) CVs at different scan rates, (**c**) charge/discharge curves (CDs) at different current densities, and (**d**) computed gravimetric and areal capacitances at different current densities.

Any material’s capacitive behavior can be measured using cyclic voltammetry. CV was performed on a symmetric supercapacitor cell made in a sandwich-type geometry utilizing TNR/CNT electrodes, paper as a separator, and 1 M H_2_SO_4_ as an electrolyte to study the capacitive properties of TNR/CNTs, and the results were compared to those of CNTs. Figure 3a shows the cyclic voltammograms produced for TNRs/CNTs and CNTs at 10 mV s^−1^. Figure 3a shows the electrodes’ outstanding stability over the applied voltage range of 0 to 1 V. Using the formula [30], the specific capacitance was determined from the voltammograms.

(2) $$C_{s}=\frac{2\times i}{m\times v}$$

where C_s_ is the specific capacitance found from the CV; ‘***i***’ is the average current found from the anodic and cathodic curves; v gives the scan rate; and ***m*** is the weight of the active material in one electrode. The CNTs’ cyclic voltammograms are quasi-rectangular along the x-axis, with a small redox peak, in the beginning, showing that they have both double-layer and pseudocapacitance behavior. Because of the widespread oxidation of CNTs, oxidizable groups such as hydroxyl, carbonyl, and carboxyl are formed at defects in the nanotube carbon lattice, resulting in pseudocapacitance [30]. However, when TiO_2_ and the CNTs worked together, the TNR/CNTs showed an improved rectangular background and oxide peak.

The TNR/CNTs performance was also assessed at various scan rates; Figure 3b shows the fluctuation of C_s_ with the scan rate. Because the redox reactions in TNR are dependent on the insertion–deinsertion of dopant ions from the electrolyte, the C_s_ values fall as the scan rate increases [31,32]. At low scan rates, ions from the electrolyte can move into almost all of the material’s pores. This causes a complete insertion reaction and the most capacitive behavior possible.

The CD curves of the TNR/CNT electrodes at different scan rates are shown in Figure 3c; the divergence from linearity is due to the pseudocapacitance from TNR. The longer charge and discharge times are due to the use of EDLC and faradic capacitance from TNRs and CNTs, respectively.

Figure 3d illustrates the supercapacitor’s gravimetric (*C_wt_*) and areal (*C_A_*) capacitances at various current densities, as derived from the charge/discharge curves using the following equations [33,34].

(3) $$C_{wt}=\frac{4I}{m\left(\frac{\Delta E}{\Delta t}\right)}$$

(4) $$C_{A}=\frac{4I}{A\left(\frac{\Delta E}{\Delta t}\right)}$$

where “*I*” represents the applied constant current (*A*); $\left(\frac{\Delta E}{\Delta t}\right)$ is the slope of the discharge curve; the two electrodes’ footprint area (cm^2^) is marked by *A*; and the total mass of the two electrodes is given by m (g).

At a current density of 0.2 A g^−1^, the gravimetric capacitance in H_2_SO_4_ reached its maximum value of 33.33 F g^−1^. As the current density decreased, the device’s area capacitance increased from 66.66 mF cm^−2^ to 80 mF cm^−2^. Choosing an electrolyte mixture improved the performance of our electrodes.

### 3.3. The TNR/CNTs Electrode’s Superior Performance

**Figure 4:**
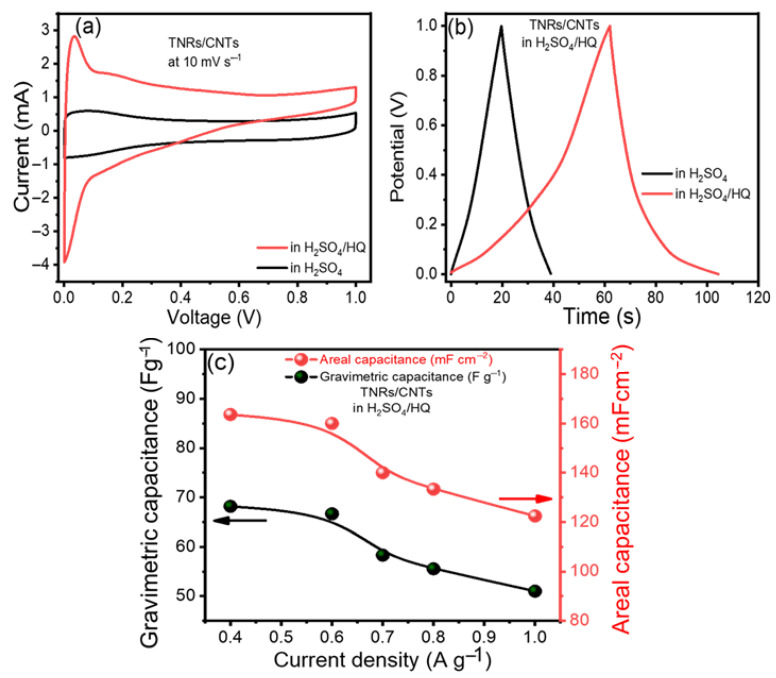
Electrochemical performance improvement: (**a**) cyclic voltammograms (CVs) and (**b**) charge/discharge curves (CDs) of TNR/CNTs in H_2_SO_4_ and a mixed electrolyte, respectively, and (**c**) gravimetric and areal capacitances of the TNR/CNTs in the mixed electrolyte at different current densities.

Hydroquinone (HQ) was added to 1 M H_2_SO_4_ to make a mixed electrolyte since it was believed to be an effective redox-active electrolyte that provided additional redox reactions [35]. Using a mixed electrolyte significantly enhanced the cyclic voltammetry integrated area (Figure 4a). In Figure 4b, the electrolyte had the longest CD duration, and the fact that it did not look like a triangle suggests that HQ is involved.

In Figure 4c, the gravimetric and areal capacitances (*C_wt_* and *C_vol_*) of the TNR/CNT electrodes in the mixed electrolyte (H_2_SO_4_/HQ) at various current densities are displayed, with *C_wt_* = 68.18 A g^−1^ demonstrating significantly superior performance than H_2_SO_4_, which was equal to 33.33 A g^−1^. Similarly, the areal capacitance per footprint of the device increased from 80 mF cm^−2^ in H_2_SO_4_ to 163.63 mF cm^−2^ in H_2_SO_4_/HQ. As a result, the development of supercapacitors with our composite TNR/CNT appears promising.

**Figure 5:**
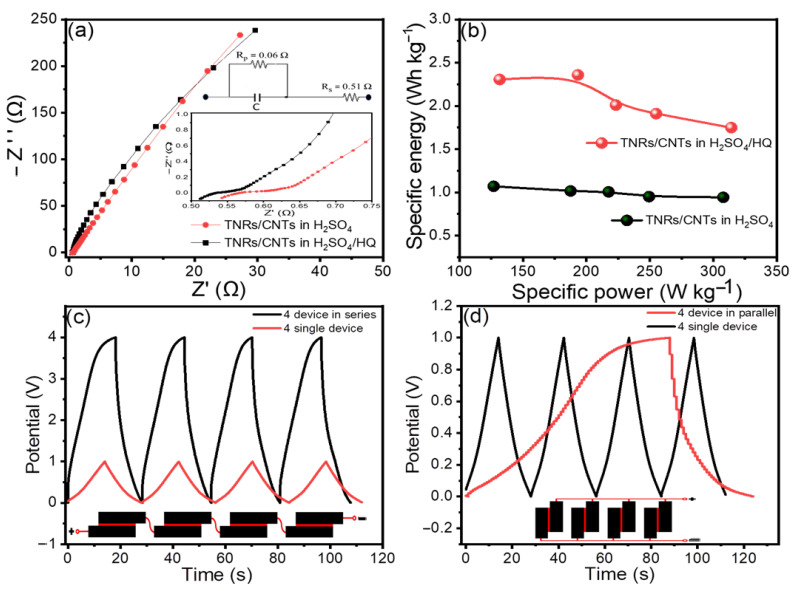
(**a**) TNR/CNT Nyquist plots in H_2_SO_4_ and H_2_SO_4_/HQ, respectively. The inset shows enhanced Nyquist plots in the high-frequency area; (**b**) a Ragone plot of specific power vs. specific energy for TNR/CNTs in H_2_SO_4_ and H_2_SO_4_/HQ, respectively; and CD curves of TNR/CNTs in H_2_SO_4_/HQ for a single cell and tandem cells at 1.9 A g^−1^ (**c**) in series, and (**d**) in parallel.

EIS measurements were taken at 100 kHz to 10 mHz, which is the frequency range. These EIS measurements aided in understanding the resistive components of the supercapacitors. In EIS, Nyquist plots are useful for determining the resistivity of the electrodes. In theory, Figure 5a is divided into two regions: a high-frequency 45-degree semicircular arc and a low-frequency straight line. The charge-transfer limiting process is represented by the high-frequency arc, in which the solution resistance (R_s_) is connected in series with the double-layer capacitance (C_dl_), which is connected in parallel with the charge-transfer resistance (R_ct_) and pseudocapacitance (C_p_). The inset of Figure 5a shows the equivalent circuit provided with the values of their elements for TNR/CNTs in H_2_SO_4_/HQ. The resistance faced by the ions as they go through the separator toward the electrode surface is known as equivalent series resistance (ESR), and it may be measured at high frequency by the first intersection point on the x-axis [36]. Based on the experimental observations, as shown in the inset of Figure 5a, the ESR values for the material in H_2_SO_4_ and (HQ + H_2_SO_4_) were 0.54 Ω and 0.51 Ω, respectively. The second intersection point on the x-axis shows the charge-transfer resistance (R_ct_), which is based on the length of the semicircular arc and is equal to the overall resistance given by the electrode/electrolyte interfaces [37]. Furthermore, R_ct_ is solely determined by the amount of active surface area on the electrode that the electrolyte can access. The length of the semicircle shown on the real axis can be used to determine R_ct_. R_ct_ values for MWCNTs and TNR/CNTs were found to be 0.1 Ω and 0.06 Ω, respectively. Due to the redox behavior of the HQ, the TNRs/CNTs had a lower charge-transfer resistance, which was the same as a lower ion diffusion resistance and a higher capacitance.

Based on the total mass of electroactive materials in the two electrodes, we constructed the Ragone plot in Figure 5b with the specific energy (*E_wt_*) and specific power (*P_wt_*) [38].

(5) $$E_{wt}\left({\mathrm{Wh}\;\mathrm{Kg}}^{-1}\right)=\frac{\;0.125\;C_{wt}{\left(\Delta E\right)}^{2}}{3.6}$$

(6) $$P_{wt}\left({W\;\mathrm{Kg}}^{-1}\right)=\frac{3600\;E_{wt}}{\Delta t}$$

The average specific energy and specific power values for the electrodes in H_2_SO_4_ were 1.14 Wh Kg^−1^ and 307.82 W Kg^−1^, respectively. When H_2_SO_4_/HQ was used as an electrolyte, much higher values of 2.35 Wh Kg^−1^ and 314.17 W Kg^−1^ were produced.

In general, a single supercapacitor’s overall energy storage capacity is insufficient for most practical applications. For a specific application, a ‘bank’ of supercapacitors with a specific voltage and capacitance rating must be linked in series or parallel. Figure 5c,d shows the adaptability of the TNR/CNT electrodes for serial and parallel combinations by linking four devices in series and parallel configurations. This is conceivable due to the tandem TNR/CNT electrode’s excellent capacity and operational voltage window being able to be controlled. Like individual supercapacitors, the tandem devices featured nearly perfect triangular charge and discharge curves, indicating good capacitive properties. This great performance was made possible without voltage balancing, which is often used with series connections to keep any cell from going into overvoltage.

**Figure 6:**
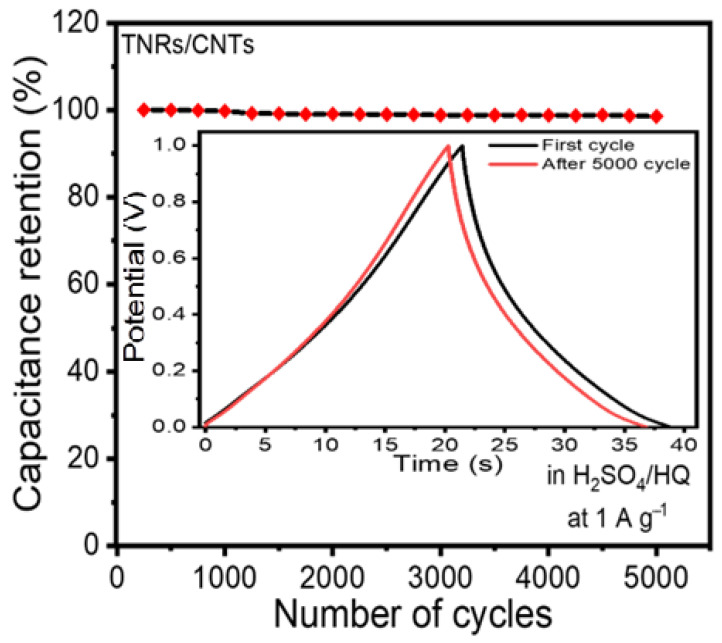
Stability of the electrode’s performance. The electrode retained 95.5 percent of its initial capacitance after 5000 cycles. The inset shows the first and last cycle for the charge/discharge curves.

The cycling performance of the cell was evaluated over 5000 cycles. The dependence of the obtained specific capacitance on the cycle number is plotted in Figure 6. Therefore, if the capacitor starts with a value of 51.02 F g^−1^ at 1 A g^−1^, only a 2.5% loss in the capacitance is observed after about 5000 cycles. This study’s cycle stability was higher than that of the other TiO_2_-based materials [7,8,9,10,11,12,13,14,15,39,40]. For example, after 3000 cycles, the capacitance of the TiO_2_ nanocrystal in 1 M KOH electrolyte was reduced by approximately 19.4% and that of the ID TiO_2_ nanotube in 1 M KOH electrolyte was reduced by approximately 33% [39,40]. The reason for this is that adding CNTs to pure TiO_2_ in our electrode substantially improved the loss in capacity.

## 4. Conclusions

Chemical vapor deposition was used after the hydrothermal method to create titanium dioxide nanoribbons (TNRs) and multi-walled carbon nanotubes (MWCNTs). The morphological characterization proved that the MWCNTs were grown in the porous pits on the surface of one phase of the TiO_2_-B nanoribbons to create a network-like structure in the nanocomposite. TNR/CNTs were used as the electrode, and H_2_SO_4_ or (H_2_SO_4_/HQ) as the electrolyte, resulting in a supercapacitor with high specific capacitance, a long cycle life, a small self-discharge process, and a high energy and power density. The TNR/CNT produced a gravimetric capacitance of 33.33 F g^−1^ in a two-electrode supercapacitor arrangement, which was increased to 68.18 F g^−1^ in a redox-active electrolyte of H_2_SO_4_/HQ. With 97.5% capacitor retention after 5000 cycles, the TNR/CNT supercapacitor also exhibited excellent cyclic stability. These findings suggest that the TNR/CNT supercapacitor may have an impact on the functionality of upcoming portable energy storage technologies.

## Figures and Tables

**Table 1:** The CNTs and nanocomposite’s crystallite sizes (CS), d-spacing, dislocation densities, texture coefficients, and strain.

		CNTs				TNRs/CNTs					
						CNTs			TiO_2_—B		
Peaks	2ϴ (Deg)	26.32	42.91	44.94	65.74	26.35	44.05	53.48	33.0020	47.7603	67.0194
	Planes	(002)	(100)	(101)	(004)	(002)	(101)	(004)	(310)	(−512)	(711)
CS (Å)		146.78	153.56	177.04	106.52	168.03	154.18	63.74	99.09	156.31	113.98
d-spacing (Å)		3.386	2.108	2.017	1.705	3.383	2.056	1.713	2.714	1.904	1.396
Dislocation density (δ) × 10^−5^ (Å^−2^)		4.64	4.24	3.19	8.81	3.54	4.21	24.61	10.18	4.09	7.70
Texture Coefficient (TC)		2.538	0.442	0.895	0.125	2.275	0.562	0.163	0.875	1.579	0.546
Microstrain only (%)		1.154	0.686	0.570	0.800	1.006	0.667	1.344	1.370	0.609	0.6126

## Data Availability

Not applicable.
